# Structural Build-Up and Stability of Hybrid Monoglyceride–Triglyceride Oleogels

**DOI:** 10.3390/gels10100650

**Published:** 2024-10-11

**Authors:** Kato Rondou, Antonia Dewettinck, Koen Dewettinck, Filip Van Bockstaele

**Affiliations:** 1 Food Structure and Function Research Group, Department of Food Technology, Safety and Health, Faculty of Bioscience Engineering, Ghent University, 9000 Ghent, Belgium; 2 Vandemoortele Centre ‘Lipid Science and Technology’, Faculty of Bioscience Engineering, Ghent University, 9000 Ghent, Belgium

**Keywords:** fat crystallization, hybrid oleogels, monoglycerides, X-ray scattering, rheology, phase contrast microscopy

## Abstract

Oleogelation is an alternative oil structuring route to formulate (semi-)solid fats with a reduced amount of saturated fats. Monoglycerides have been identified as effective gelators; however, their application potential can be limited due to challenges regarding mechanical strength and long-term stability. Therefore, the formulation of hybrid fat blends is a promising way to improve the functionality of oleogels. This research focuses on the interaction between mono- and triglycerides (MAGs and TAGs) in hybrid oleogels. A total gelator concentration of 10% (*w*/*w*) with changing MAGs–TAGs ratios (increase by 25% on a molar basis; M0-T100, M25-T75, M50-T50, M75-T25, M100-T0) was used. First, the oleogels were produced without shear to unravel the crystallization behavior (DSC, SAXS, WAXS). Next, the oleogels were crystallized with shear to assess the interactions between MAGs and TAGs on macroscale properties (rigidity, oil binding capacity) during storage of 1 day, 1 week, and 4 weeks. A clear distinction could be made between the MAG crystals and TAG crystals in the blends M50-T50 and M75-T25 based on WAXS, SAXS, and phase contrast microscopy. This indicates that both gelators crystallize separately. During the follow-up study of the dynamically produced samples, a synergistic effect was found for Dy-M50-T50 and Dy-M75-T25; however, it was not maintained upon storage. The initial rigidity of 2.4 × 10^4^ Pa and 2.0 × 10^4^ Pa decreased to 1.5 × 10^4^ Pa and 1.0 × 10^4^ Pa for Dy-M50-T50 and Dy-M75-T25, respectively.

## 1. Introduction

The increasing demand for healthier and sustainable food products has driven the food industry to investigate alternatives. Related to the fat content, oleogelation is an interesting approach in which liquid oils are structured using gelators. Unlike traditional solid fats, oleogels have a reduced saturated fat content while still providing structure and stability. This replacement of saturated fats with unsaturated fats results in improved cardiovascular health [1,2,3]. Different structuring routes can be applied, namely crystalline particles, polymer networks, and self-assembled networks. Regarding the crystalline particles, the structuring mechanism is similar compared to conventional fat structuring with high melting triglycerides. Examples of this type of gelators are monoacylglycerols (monoglyceride; MAG), diglycerides, waxes, … [2,4]. Hereby, one of the promising approaches is the use of MAGs. The application potential of MAG-based oleogels is summarized in a review by Palla et al. (2022) [5]. This review clearly evaluates research on many food products in which traditional fats were replaced by oleogels. The variety of food applications, ranging from bakery applications and confectionaries to meat-based products, illustrates the high application potential. Nevertheless, limitations related to mechanical strength and long-term stability might hamper the application in the food industry [6].

To overcome the limitations of MAG-based oleogels, hybrid fat blends offer a promising solution to improve the structure and stability. Synergistic effects have been observed when combining MAGs with various other gelators, such as waxes [7,8,9], phytosterols [10,11], and ethylcellulose [12]. Important to note is that the final outcome is largely affected by the formulation and processing conditions, where the interaction between the different gelators plays a key role in determining the overall behavior. Within this research, a combination of hydrogenated triacylglycerols (triglyceride; TAGs) and hydrogenated MAGs is used. Both gelators can be used individually to structure liquid oil, each with a different crystallization behavior on the nanoscale [2]. For TAGs, the most common polymorphs are the α, β′, and β polymorphs, while these are Lα, sub-α, and β for MAGs. It is known that MAGs influence the crystallization behavior of TAGs [13,14,15]; however, their synergetic effects on oil structuring were not widely investigated. To the best of our knowledge, this was only investigated by da Silva and Danthine (2021), where static crystallization of palm-based MAGs with fully hydrogenated rapeseed oil (total gelator concentration of 6%) in rapeseed oil did not yield a synergistic effect.

This research focuses on the structural build-up and stability of hybrid fat blends containing high melting MAGs and TAGs. The research will be conducted in two parts: first, the crystallization behavior of oleogels will be investigated upon static crystallization (i.e., without shear). Subsequently, the oleogels crystallized with a lab-scale scraped surface heat exchanger will be evaluated over different storage periods (1 day, 1 week, and 4 weeks). These processing conditions are relevant for industrial processing and enable further investigation of macroscopic properties such as rigidity and oil binding capacity as functions of the storage time [16]. A combination of both parts is necessary to better understand the behavior of hybrid oleogels and their application potential.

## 2. Results and Discussion

### 2.1. Crystallization and Melting Behaviors

#### 2.1.1. Impact of Cooling Rate

##### Thermal Properties

**Figure 1:**
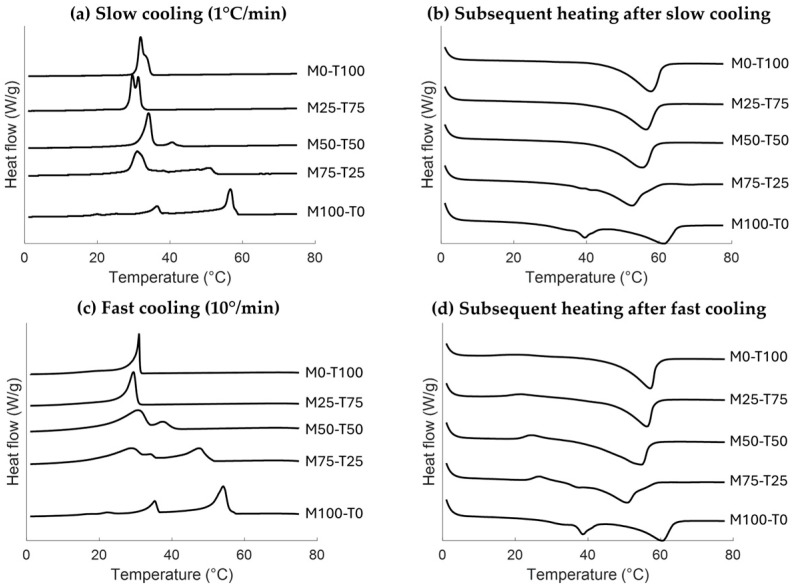
Crystallization curves (**a**–**c**) upon cooling at 1 °C/min (**a**,**b**) and 10 °C/min (**c**,**d**) and the following melting curves (**b**–**d**) when heating at 10 °C/min.

The crystallization and melting behavior of the references, namely the oleogels containing only TAG (M0-T100) and MAG (M100-T0) in rapeseed oil and the hybrid oleogels (M25-T75, M50-T50, M75-T25), are analyzed with differential scanning calorimetry (DSC). Combining the two hardstocks results in a complex crystallization behavior. Figure 1 shows the crystallization and melting curves of both the slow- and fast-cooled samples. It can be observed that the applied cooling rate had a major influence on the crystallization and melting curves for TAG-containing samples. For the slow-cooled M0-T100, a shoulder can be observed, while the fast-cooled samples only showed one sharp peak. Based on the literature and the relatively pure composition (Table S1), this shoulder is expected to be related to a polymorphic transition from the α polymorph to the β polymorph [17]. The effect of the cooling rate was less evident for M100-T0. The crystallization behavior for both cooling rates was as described in the literature, namely, first, the Lα polymorph is formed, followed by a polymorphic transition towards sub-α1, and finally to sub-α2 [18,19,20]. With a decreasing number of MAGs in the blends, the crystallization peak related to Lα could be distinguished until the 50:50 ratio (M50-T50). Hereby, a decrease in onset temperature of 20 °C from M100-T0 towards M50-T50 was observed for both cooling rates (Table 1). After the Lα crystallization, the following crystallization peak(s) can be the result of a polymorphic transition of the MAG fraction or the crystallization of the TAG fraction. More insights into these events will be obtained with X-ray scattering in the following part. For M25-T75, it is expected that the crystallization peak is mainly the TAG crystallization. When comparing M25-T75 with M0-T100, a small decrease in the onset temperature of crystallization could be observed (Table 1, Figure S1). Similarities between TAGs and MAGs can cause attractive interactions that prevent the formation of nuclei at lower MAG concentrations. However, this decrease in temperature is not significant. Even though all the oleogels contain a total hardstock concentration of 10% (*w*/*w*), a higher total crystallization enthalpy was found for M100-T0 compared to M0-T100. Also, for the MAG-containing blends, a higher crystallization enthalpy could be observed for M50-T50 and M75-T25 compared to M25-T75 (Table 1, Figure S2).

Upon heating, the endothermic peaks of the slow- and fast-cooled oleogels are very similar. Yet, an additional small exothermic peak around 20–30 °C can be observed for the fast-cooled TAGs containing oleogels. Lower cooling rates and/or smaller undercooling conditions are required for direct crystallization towards the more stable polymorphs [21]. As a result, the cooling step at 10 °C/min until 0 °C might be ineffective to initiate the polymorphic transition towards the β polymorph. During the subsequent heating step, the increase in energy might be sufficient for the polymorphic transition from α to β of the TAGs present.

##### Polymorphism

**Figure 2:**
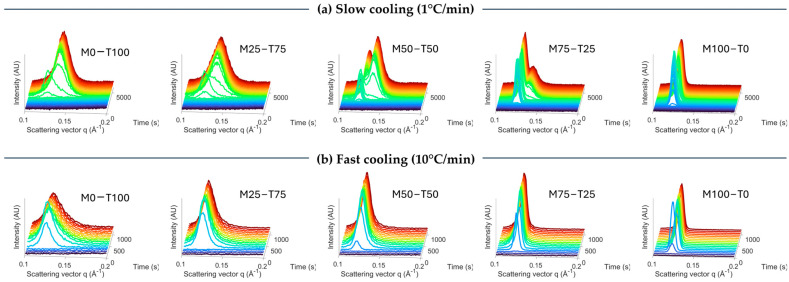
Evolution of the SAXS profile during crystallization from 80 °C to 0 °C at 1 °C/min (**a**) and 10 °C/min (**b**). The color gradient from blue to red corresponds to the crystallization time, going from the start of the cooling step towards the end of the isothermal time at 0 °C.

The differences in crystallization behavior were further investigated with X-ray scattering. Hereby, the evolution of the first-order SAXS peak clearly illustrates the effect of the cooling rate (Figure 2). Important to note is that the acquisition time was kept constant at 60 s, resulting in more measurements when cooled at 1 °C/min. Starting with M0-T100 cooled at 1 °C/min, it can be observed that first, a small peak with a d-spacing of 50.2 Å appears, followed by a fast shift to 45.0 Å. The polymorphs were identified based on the WAXS profile (Figure S3). First, the α polymorph is formed with the characteristic d-spacing of 4.07 Å. Second, a polymorphic transition towards β occurred, characterized by d-spacings 4.47, 3.78, and 3.61 Å. This was in line with the literature [17,22]. Remarkably, the SAXS profile of M0-T100 cooled at 10 °C/min showed only a minor shift from 50.5 Å to 49.8 Å. This indicates an incomplete transition, which is confirmed by the WAXS profile (Figure S3) and the results obtained with DSC (part thermal properties). At the end of the isothermal time, the small broad peak around 4.44 Å is still present, while an additional small peak at 4.44 Å and two weak bumps around 3.80 and 3.63 Å appeared upon crystallization.

For M100-T0 cooled at 1 °C/min, first, a peak appears at 51.1 Å, followed by a transition towards 49.6 Å, and finally to 50.0 Å. The polymorphs are, respectively, Lα (4.11 and 4.08 Å), sub-α1 (4.13 Å and three peaks in 3.86–5.57 Å), and sub-α2 (4.05 Å and three peaks between 3.79 and 3.50 Å). These transitions were also described in the literature [18,23]. The first polymorphic transition of Lα to sub-α1 is clearly visible in SAXS, while this is not the case for the sub-α1 to sub-α2 transition due to relatively small shifts. The same transitions occurred when cooling at 10 °C/min.

**Figure 3:**
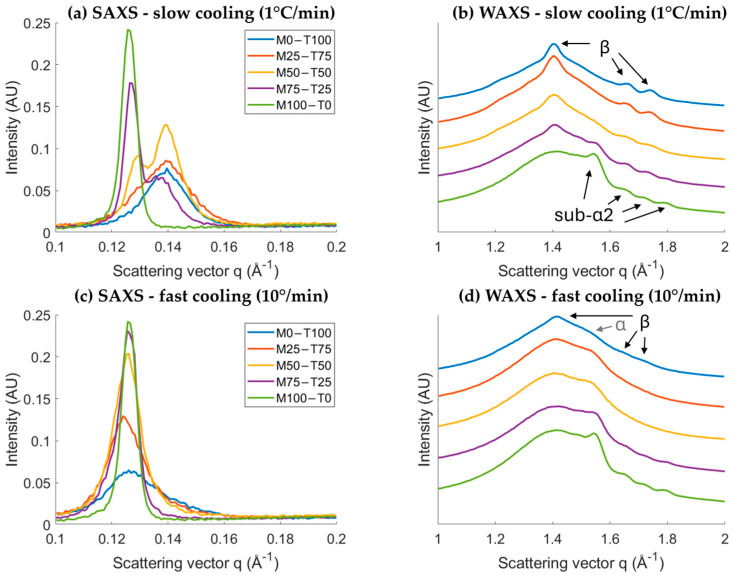
Final SAXS (**a**–**c**) and WAXS (**b**–**d**) spectra at the end of the isothermal time at 0 °C upon crystallization at 1 °C/min (**a**,**b**) and 10 °C/min (**c**,**d**).

Combining TAGs and MAGs resulted in the formation of two co-existing SAXS peaks for M50-T50 and M75-T25 upon cooling at 1 °C/min (Figure 2a). To further investigate the position of these peaks, the SAXS profiles of the different samples obtained at the end of the isothermal time are shown in Figure 3a. This figure demonstrates that the first peak occurs in the same q-range as the peak of M100-T0, and the second peak appears within the range of M-T100. More specifically, the final SAXS d-spacings of M50-T50 and M75-T25 were, respectively, 45.0 Å + 48.5 Å and 45.7 Å + 49.7 Å. Together with the final WAXS profiles in Figure 3, it can be concluded that two different crystals are being formed during crystallization at 1 °C/min for M50-T50 and M75-T25. The TAGs and MAGs are, respectively, present in the β and sub-α2 polymorphs. Also, for M25-T75, a shoulder in the region 48–50 Å is observed, which might indicate the presence of both TAG-β and MAG-sub-α2. However, the MAG concentration was too low to confirm this with the used equipment. Similar to M0-T100, the TAG polymorphic transitions towards the β polymorph were limited or absent for the different blends when cooled at 10 °C/min (Figure 3). As a result, the TAGs-α polymorph remains present for all the samples containing TAGs when cooled at 10 °C/min.

**Figure 4:**
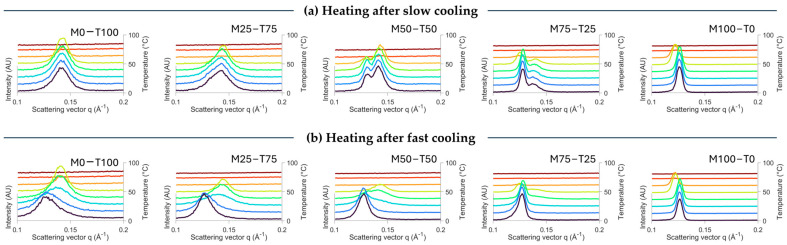
Evolution of the SAXS spectrum upon melting (from blue to red; 0°C to 80°C) at 10 °C/min after being crystallized at 1 °C/min (**a**) or 10 °C/min (**b**) until 0 °C followed by an isothermal time of 10 min.

As mentioned before, the exothermic peak around 20–30 °C in the melting profile of TAG-containing blends could be related to a polymorphic transition (Figure 1d). To confirm this, the SAXS spectra during melting are shown in Figure 4. Melting of the slowly cooled blends resulted in a decrease in intensity until the sample was completely molten without shifts. The only exception is M100-T0, for which it is known that the same polymorphic transitions occur during the subsequent heating step [19]. However, upon heating the fast-cooled blends, the SAXS peak related to the β polymorph starts to develop. This indicates that fast cooling to a temperature far below the crystallization temperature of TAGs hinders the formation of the β polymorph, while subsequent heating allows further transition [24].

#### 2.1.2. Impact of the Final Temperature

**Figure 5:**
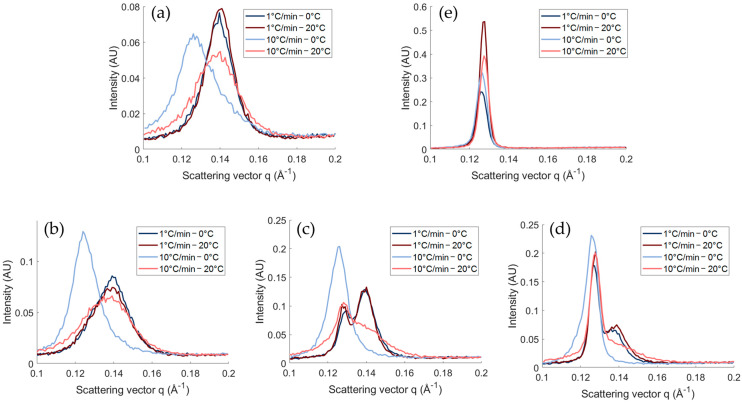
Final SAXS spectrum at the end of the isothermal time at 0 °C and 20 °C upon crystallization at 1 °C/min and 10 °C/min for (**a**) Dy-M0-T100, (**b**) Dy-M25-T75, (**c**) Dy-M50-T50, (**d**) Dy-M75-T25, and (**e**) Dy-M100-T0.

Fast cooling to a low temperature seemed to affect the crystallization behavior of TAGs. In Figure 5, the effect of decreasing the degree of supercooling by increasing the final temperature is visualized. The change in temperature did not affect the SAXS profile for the slow-cooled samples. For the fast-cooled samples, the SAXS profiles of the samples cooled to 20 °C are similar compared to all the slow-cooled samples. This is also confirmed by the WAXS profiles (Figure S4), where only the fast-cooled TAG-containing samples until 0 °C showed a deviating WAXS spectrum. The increase in temperature from 0 °C to 20 °C provided the TAGs with sufficient energy to facilitate the polymorphic transition towards the β polymorph during fast cooling. This confirms the fact that lower cooling rates and/or smaller supercooling conditions result in fast transitions toward the more stable polymorphs [21].

#### 2.1.3. Crystal Morphology

**Figure 6:**
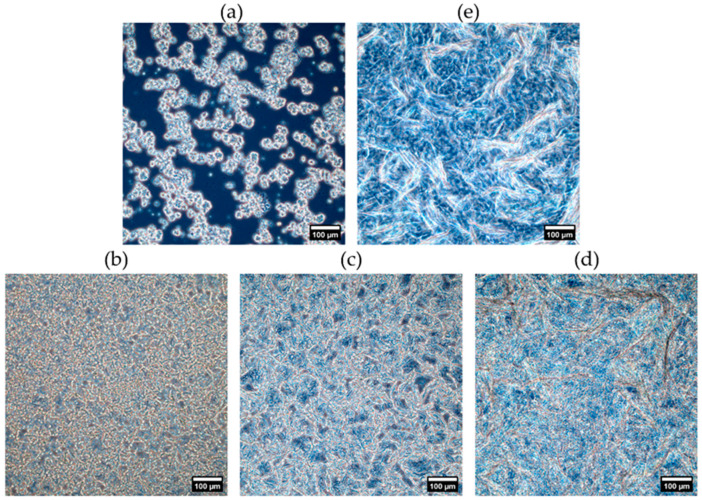
Phase contrast microscopy images of the statically crystallized oleogels ((**a**) Dy-M0-T100, (**b**) Dy-M25-T75, (**c**) Dy-M50-T50, (**d**) Dy-M75-T25, (**e**) Dy-M100-T0) after crystallization at 1 °C/min until 20 °C.

The microstructures after crystallization at 1 °C/min until 20 °C are visualized in Figure 6. Hereby, major differences between the reference samples M0-T100 and M100-T0 can be observed. The fat crystals of M0-T100 are more rounded and smaller compared to the long, elongated fat crystals of M100-T0. In the blends M50-T50 and M75-T25, both types of fat crystals can still be distinguished. Similar to the SAXS and WAXS results, less pronounced differences were found for M25-T75, given the low MAG concentration.

### 2.2. Stability of Dynamically Produced Oleogels

#### 2.2.1. Melting Behavior

**Figure 7:**
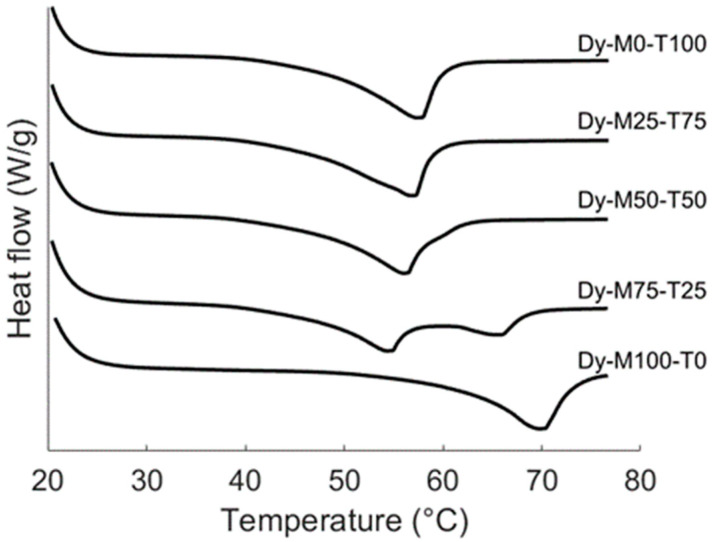
Melting curves (10 °C/min) of the dynamically produced oleogels after 1 day of storage at 20 °C (week 0).

The previous part characterized the fat crystal network of statically produced oleogels. Under dynamic conditions, the oleogel blends are crystallized under shear (in an SSHE) and fast cooling rate. Such conditions are more relevant for industrial processing and are known to strongly impact the resulting fat structure [25]. Figure 7 shows the melting curves of the dynamically produced oleogels after 1 day of storage. Hereby, the oleogels Dy-M0-T100 and Dy-M25-T75 still showed only one clear melting peak. Nonetheless, for Dy-M50-T50, a small shoulder was present at the right side of this melting peak. Contrarily to the melting profile of statically produced M100-T0, the melting profile of Dy-M100-T0 only showed one peak instead of three. This indicates that the MAGs are present in the β polymorph. The more stable β polymorph also has a higher melting point compared to the Lα polymorph [19]. Dy-M75-T25 showed two separated crystallization peaks, the first one related to the melting of the TAG fraction and the second one related to the melting of the MAG fraction, both in the β polymorph. No major changes occurred in melting temperature during storage for all the oleogels (Table S2). These results indicate that applying shear during crystallization enhances the polymorphic transition toward the most stable β polymorph for the MAGs.

#### 2.2.2. Crystal Morphology

**Figure 8:**
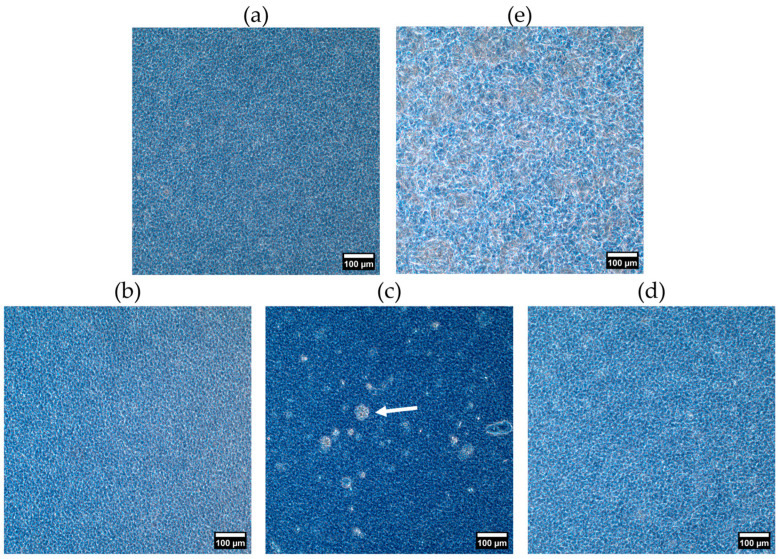
Phase contrast microscopy images of the dynamically produced oleogels ((**a**) Dy-M0-T100, (**b**) Dy-M25-T75, (**c**) Dy-M50-T50, (**d**) Dy-M75-T25, (**e**) Dy-M100-T0) after 1 day of storage at 20 °C (week 0).

The application of shear during crystallization resulted in a homogeneous crystal network with smaller crystals (Figure 8) compared to static crystallization (Figure 6). Shear may provide extra energy to overcome the activation energy to start the nucleation. As a result, the nucleation rate increases, and smaller crystals are formed [26]. Only for Dy-M50-T50, small aggregates (see arrows) were observed within the network after one day of storage. Nonetheless, the formation of small aggregates was found for M0-T100 and M25-T75 upon storage (Figures S5–S9). For Dy-M100-T0, the crystals still have a more elongated shape compared to Dy-M0-T100; however, this is less pronounced than for the static oleogels. Additionally, the differences in microstructure between Dy-M25-T75, Dy-M50-T50, and Dy-M75-T25 are less clear.

#### 2.2.3. Physicochemical Properties

**Figure 9:**
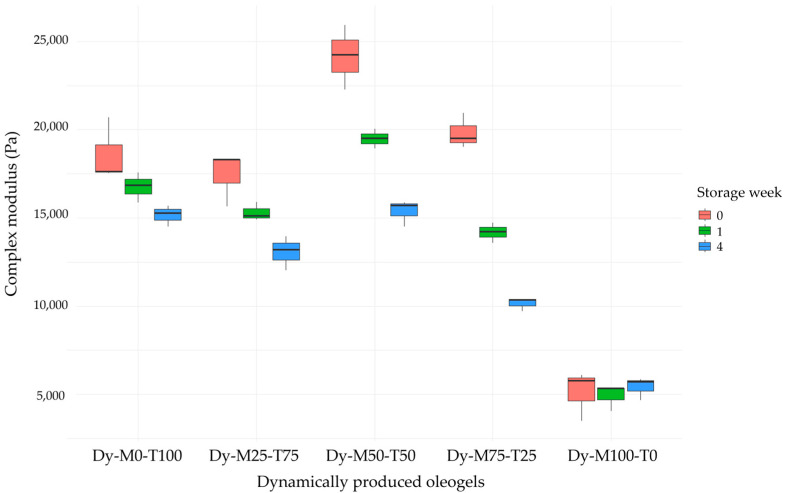
Complex modulus in the linear viscoelastic region of the dynamically produced oleogels as a function of the storage time. The boxplots represent the distribution of the complex modulus across three repetitions for each sample at the given time points (week 0, week 1, week 4).

The application potential of oleogels is largely dependent on their physicochemical properties. The rigidity, the resistance to shear, and the oil binding capacity are of main importance. The rigidity is expressed in terms of the complex modulus (Figure 9, Table 2). One day after production, the highest rigidity was found for the blends Dy-M50-50 and Dy-M75-T25 and the lowest for Dy-M100-T0. This indicates the formation of a stronger network when combining MAGs with TAGs. However, the complex modulus largely decreased as a function of the storage time for the blends (Figure 9). Also, for the flow point, which is related to structure breakdown, the highest value was found for Dy-M50-T50 after 1 day of storage, while it decreased upon storage. The overall viscoelasticity was represented by means of the phase shift angle. A phase shift angle of 0° corresponds to an ideal elastic material, while a value of 90° corresponds to an ideal viscous material. For all the oleogels, the phase shift angle was between 6 and 16°, indicating a predominant elastic behavior at small deformations. These results indicate the high potential of combining TAG and MAG hardstocks in terms of network formation; however, these systems do not maintain their structure upon storage. Based on Figures S5–S9, no crystal aggregation was observed for Dy-M50-T50 and Dy-M75-T25, so the decrease in network rigidity might be the result of molecular interactions. Hereby, storage at a lower temperature might be needed to maintain its structure. Nevertheless, high values for the oil binding capacity were found for all oleogels during the full storage time of 4 weeks. Overall, it can be observed that Dy-M100-T0 is the least favorable oleogel because of the lower rigidity, resistance to shear, and oil binding capacity. This could be linked to its crystal morphology, where bigger, more elongated crystals were present (Figure 8e) [27].

## 3. Conclusions

The crystallization behavior and stability of hybrid oleogels containing different ratios of MAGs and TAGs were investigated. Applying a fast cooling rate (10 °C/min) inhibited the formation of the β polymorph of the TAGs. Regarding the slow-cooled samples (1 °C/min), a clear distinction between the MAG crystals in the sub-α2 polymorph and the TAG crystals in the β polymorph could be made. The follow-up study of the dynamically produced oleogels showed that combining MAGs and TAGs (Dy-M50-T50 and Dy-M75-T25) resulted in increased rigidity and flow stress, while this largely decreased as a function of the storage time. The lowest rigidity, resistance to shear, and oil binding capacity were found for Dy-M100-T0, illustrating the limitations related to mechanical strength. These results indicate the potential of hybrid oleogels; however, long-term stability should be improved to maintain the synergistic effect.

## 4. Materials and Methods

### 4.1. Samples

The fully hydrogenated triglyceride and monoglyceride hardstocks were kindly provided by Vandemoortele Lipids NV (Izegem, Belgium). Both hardstocks originated from rapeseed oil, resulting in a high C18:0 concentration (90.5% for TAGs and 90.8% for MAGs) and a low amount of C16:0 (5.3% for both hardstocks) (Table S1). Rapeseed oil was purchased from Ranson (Harelbeke, Belgium).

### 4.2. Oleogel Production

The oleogels were prepared by mixing the hardstocks on a molar basis, ranging from 100% TAGs (M0-T100) to 100% MAGs (M100-T0) in steps of 25%. The total hardstock concentration in the oleogels was 10% on a weight basis. The fat blends were heated to 80 °C to obtain a homogeneous solution. The crystallization step was performed using two different methods. The first crystallization method was static production, in which the samples were directly poured into 125 mL containers and left to crystallize at room temperature without stirring. The resulting oleogels M0-T100, M25-T75, M50-T50, M25-T75, and M100-T0 were stored at 20 °C. The second method included a lab-scale scraped surface heat exchanger (AC-unit; Het Stempel, Zwijndrecht, The Netherlands) [18]. The homogeneous solution at 80 °C was pumped at 20 rpm through a scraped surface heat exchanger operating at 1000 rpm, and the temperature of the water bath was set at 5 °C. This resulted in a flow rate of 43.2–48.0 g/min with an outlet temperature between 11.7 and 13.0 °C. After sampling, the oleogels were stored at 20 °C, and the samples will be referred to as Dy-M0-T100, Dy-M25-T75, Dy-M50-T50, Dy-M75-T25, and Dy-M100-T0. The samples were analyzed after 1 day (week 0), 1 week, and 4 weeks to investigate their stability.

### 4.3. Analysis of the Statically Produced Oleogels

#### 4.3.1. DSC

The crystallization and melting behaviors of the statically produced oleogels were investigated with differential scanning calorimetry (DSC; DSC2500—TA Instruments|Waters, New Castle, DE, USA). A small amount of sample was added to a Tzero pan and closed with a Tzero hermetic lid. The static samples were heated to 80 °C for 10 min, followed by a cooling step to 0 °C for 10 min and a heating step at 10 °C/min to 80 °C. Two different cooling rates were used for each sample, namely 1 °C/min as a slow cooling rate and 10 °C/min as a fast cooling rate. Measurements were performed in triplicate, and the results were analyzed in TRIOS (TA instruments) [28].

#### 4.3.2. X-ray Scattering

Crystal polymorphism was analyzed with a Xeuss 3.0 X-ray scattering (XRS) system (Xenocs; Grenoble, France) operating with an Eiger2R 1M detector (Dectris). The X-ray beam was generated by a Cu-source (Genix 3D) with a wavelength of 1.54 Å at 50 kV and 0.60 mA. The sample-to-detector distances for WAXS and SAXS were, respectively, 55 mm and 360 mm. A small amount of sample was added in a 1 mm borosilicate glass capillary (WJM-Glas, Berlin, Germany) and heated for 10 min at 80 °C prior to acquisition. The same time–temperature protocol as the DSC analysis was applied, which was controlled by a Linkam HFSX350-CAP system cooled with liquid nitrogen. An acquisition time of 60 s was used for both SAXS and WAXS. The spectra were corrected by subtracting the spectrum of an empty capillary.

#### 4.3.3. Phase Contrast Microscopy

The microstructure was visualized with phase contrast microscopy (PCM) (Leica DM2500 LED, Machelen, Belgium). A small amount of sample was added to the microscope slide, gently covered with the coverslip, and heated to 80 °C for 10 min. Crystallization was followed by using both slow cooling (1 °C/min) and fast cooling (10 °C/min) until 20 °C. A 10× magnification was used (HC PL FLUOTAR 10×/0.32 PH1), and images were taken with LAS X Time-Lapse (version 3.9.0.28093). Afterward, the contrast of the images was adapted with ImageJ (version 1.54d) based on the procedure of Campos (2012) [29].

### 4.4. Follow-Up Study of the Dynamically Produced Oleogels

#### 4.4.1. Phase Contrast Microscopy

The microstructure of the dynamically produced oleogels was visualized with phase contrast microscopy (PCM) (Leica DM2500 LED, Belgium). A small amount of oleogel was taken and visualized with a 10× magnification (HC PL FLUOTAR 10×/0.32 PH1) via the LAS X 3.9.0.28093 software. Afterward, the contrast of the images was adapted with ImageJ (version 1.54d) [29].

#### 4.4.2. Rheology

The viscoelastic properties of the dynamically produced oleogels were determined with a rheometer (AR 2000 ex—TA Instruments; New Castle, DE, USA). A sandblasted plate–plate geometry with a diameter of 25 mm and a gap of 1 mm was used. An equilibration time of 5 min was applied, after which a strain sweep with a logarithmic ramp (γ = 0.0001–2, ω = 1 s^−1^) was performed. The temperature was kept constant at 20 °C using a Peltier temperature control system. The measurements were performed in triplicate. The end of the linear viscoelastic region (LVR) was determined by a 10% deviation from the average value of the initial five data points of the storage modulus. The mean complex modulus and phase shift angle were calculated within the LVR, the yield stress at the end of the LVR, and the flow stress at the intersection of the storage and loss modulus [25,30].

#### 4.4.3. Oil Binding Capacity

The ability of the dynamically produced oleogels to hold the liquid oil was analyzed via centrifugation. A sample amount of 5 g was added to a 15 mL falcon tube and centrifuged for 30 min at 4500 rpm (ROTINA 380R—Hettich, Geldermalsen, The Netherlands) at a constant temperature of 20 °C. After centrifugation, the falcon tubes containing the oleogel was placed upside down for 1 h at room temperature to remove the unbound oil. The oil binding capacity (OBC) was calculated as $OBC=\left(1-\frac{m_{1}-m_{2}}{m_{1}}\right)\cdot 100$, with m_1_ as the mass of the tube containing the oleogel before centrifugation and m_2_ as the mass after 1 h upside down. The measurement was performed in triplicate [25,31].

### 4.5. Statistics

Statistical analysis was conducted using RStudio (version 2024.04.2). Homogeneity and normality were assessed using the Levene and Shapiro–Wilk test, respectively. Based on the outcome, the ANOVA or Kruskal–Wallis test and Tukey or DunnettT3 tests were used to compare the means. The differences in mean related to the cooling rate were assessed with paired *t*-test or Wilcoxon test based on the assumptions. All tests were performed with a significance level of 0.05.

## Figures and Tables

**Table 1:** Crystallization properties obtained with DSC: total enthalpy, onset temperature, and peak temperatures of the main events. Superscripts a–d indicate significant differences (*p* < 0.05) between the different oleogels, while superscripts A–B indicate significant differences (*p* < 0.05) between the applied cooling rate for each oleogel.

	Cooling Rate (°C/min)	Total Enthalpy (J/g)	Onset Temperature (°C)	Peak Temperature Event 1 (°C)	Peak Temperature Event 2 (°C)	Peak Temperature Event 3 (°C)
M0-T100	1	17.9 ± 0.3 ^a,A^	33.41 ± 0.08 ^a,A^	31.90 ± 0.03 ^a,A^	n/a	n/a
	10	13.4 ± 0.1 ^a,A^	31.26 ± 0.09 ^a,B^	30.99 ± 0.07 ^a,A^	n/a	n/a
M25-T75	1	17.5 ± 0.5 ^a,A^	32.51 ± 0.29 ^a,A^	31.31 ± 0.19 ^a,A^	29.75 ± 019 ^a^	n/a
	10	12.1 ± 0.4 ^a,A^	31.19 ± 0.53 ^a,A^	29.31 ± 0.47 ^a,B^	n/a	n/a
M50-T50	1	15.9 ± 0.3 ^b,A^	35.45 ± 0.07 ^b,A^	40.22 ± 0.55 ^b,A^	34.06 ± 0.07 ^b,A^	n/a
	10	10.1 ± 0.4 ^b,A^	34.13 ± 0.08 ^b,A^	36.99 ± 0.76 ^b,B^	30.73 ± 0.15 ^a,A^	n/a
M75-T25	1	15.4 ± 0.3 ^b,A^	51.41 ± 0.93 ^c,A^	49.33 ± 1.14 ^c,A^	36.45 ± 1.41 ^b,c,A^	n/a
	10	11.2 ± 1.1 ^a,b,B^	49.23 ± 1.06 ^c,B^	46.79 ± 0.60 ^c,B^	33.98 ± 0.35 ^b,A^	28.73 ± 0.32 ^a^
M100-T0	1	19.4 ± 0.1 ^c,A^	56.04 ± 1.51 ^c,A^	54.87 ± 1.53 ^d,A^	37.01 ± 0.46 ^c,A^	25.92 ± 0.58 ^A^
	10	17.9 ± 0.2 ^c,B^	53.41 ± 2.12 ^c,B^	52.65 ± 1.32 ^d,A^	36.02 ± 0.65 ^c,A^	22.83 ± 0.58 ^a,A^

**Table 2:** Physicochemical properties of the dynamically produced oleogels upon storage. Superscripts a–e indicate significant differences (*p* < 0.05) between the different oleogels, while superscripts A–C indicate significant differences (*p* < 0.05) as a function of the storage time. “*” is used in case only two repetitions were used.

	Storage (Weeks)	Complex Modulus in LVR (Pa)	Phase Shift Angle in LVR (°)	Yield Stress (Pa)	Flow Point (Pa)	OBC (%)
Dy-M0-T100	0	1.9 × 10^4^ ± 1.8 × 10^3 a,b,A^	6.07 ± 0.17 ^a,A^	26 ± 4 ^a,A^	481 ± 9 ^a,A^	99.7 ± 0.3 ^a,A^
	1	1.7 × 10^4^ ± 8.5 × 10^2 a,b,c,A,B^	6.38 ± 0.15 ^a,A^	22 ± 2 ^a,A,B^	450 ± 4 ^a,B^	99.8 ± 0.2 ^a,A^
	4	1.5 × 10^4^ ± 6.1 × 10^2 a,B^	6.36 ± 0.18 ^a,A^	18 ± 2 ^a,B^	434 ± 6 ^a,B^	99.2 ± 0.4 ^a,A^
Dy-M25-T75	0	1.7 × 10^4^ ± 1.5 × 10^3 a,A^	6.26 ± 0.27 ^a,A^	22 ± 3 ^a,A^	428 ± 3 ^b,A^	99.4 ± 0.6 ^a,A^
	1	1.5 × 10^4^ ±5.3 × 10^2 a,A,B^	6.60 ± 0.08 ^a,A^	16 ± 2 ^a,A,B^	409 ± 6 ^b,B^	100.0 ± 0.1 ^a,A^
	4	1.3 × 10^4^ ± 9.6 × 10^2 a,b,B^	6.61 ± 0.25 ^a,b,A^	12 ± 2 ^a,c,B^	382 ± 10 ^b,C^	99.8 ± 0.3 ^a,A^
Dy-M50-T50	0	2.4 × 10^4^ ± 1.8 × 10^3 b,c,A^	6.39 ± 0.07 ^a,A^	24 ± 4 ^a,b,A^	519 ± 9 ^c,A^	99.4 ± 0.2 ^a,A^
	1	1.9 × 10^4^ ± 5.6 × 10^2 b,B^	7.22 ± 0.08 ^b,A^	19 ± 2 ^a,A,B^	452 ± 5 ^a,B^	99.5 ± 0.4 *
	4	1.5 × 10^4^ ± 7.5 × 10^2 a,C^	7.47 ± 0.46 ^a,b,A^	15 ± 4 ^a,B^	419 ± 13 ^a,b,C^	99.6 ± 0.2 ^a,A^
Dy-M75-T25	0	2.0 × 10^4^ ± 1.0 × 10^3 a,c,A^	7.27 ± 0.51 ^a,A^	12 ± 5 ^b,A^	353 ± 1 ^d,A^	99.6 ± 0.4 ^a,A^
	1	1.4 × 10^4^ ± 5.7 × 10^2 a,B^	8.06 ± 0.98 ^a,b,A^	7 ± 4 ^b,A^	276 ± 8 ^c,B^	99.0 ± 0.6 *
	4	1.0 × 10^4^ ± 3.9 × 10^2 b,C^	8.18 ± 0.40 ^b,A^	7 ± 2 ^b,c,A^	234 ± 9 ^c,C^	98.7 ± 0.6 ^a,A^
Dy-M100-T0	0	5.1 × 10^3^ ± 1.4 × 10^3 d,A^	12.59 ± 2.24 ^a,A^	2 ± 1 ^c,A^	75 ± 3 ^e,A^	96.6 ± 1.3 ^a,A^
	1	4.9 × 10^3^ ± 7.6 × 10^2 c,A^	16.10 ± 2.28 ^b,A^	2 ± 1 ^b,A^	57 ± 5 ^d,B^	94.4 ± 0.8 ^b,A,B^
	4	5.4 × 10^3^ ± 6.5 × 10^2 c,A^	15.40 ± 1.35 ^c,A^	3 ± 2 ^b,A^	52 ± 4 ^d,B^	93.6 ± 0.4 ^b,B^

## Data Availability

The data presented in this study are openly available in Zenodo at https://doi.org/10.5281/zenodo.13819621.

## Supplementary Materials

The following supporting information can be downloaded at: https://www.mdpi.com/article/10.3390/gels10100650/s1, Table S1: Composition of the fully hydrogenated TAG and MAG hardstocks; Table S2: Melting temperatures of the dynamically produced oleogels upon storage; Figure S1. Onset temperature of crystallization for the slow- and fast-cooled hybrid oleogels; Figure S2. Total enthalpy of the slow- and fast-cooled hybrid oleogels; Figure S3: Evolution of the WAXS profiles upon crystallization; Figure S4: Final WAXS profiles at 0 °C and 20 °C; Figures S5–S9: PCM images upon storage of the dynamically produced oleogels.
