# Supplementary material for: Structural Build-Up and Stability of Hybrid Monoglyceride–Triglyceride Oleogels
Source: Gels. 2024 Oct 11;10(10):650. doi: 10.3390/gels10100650 (PMC11507350; doi:10.3390/gels10100650)
Supplement: Supplementary file 1 [file gels-10-00650-s001.zip › gels-3244589-supplementary.pdf]

Structural Build-Up and Stability of Hybrid Monoglyceride–Triglyceride Oleogels

Kato Rondou [1], Antonia Dewettinck [1], Koen Dewettinck [1,2], Filip Van Bockstaele [1,2,*]

[1] Food Structure and Function Research Group, Department of Food Technology, Safety and Health, Faculty of Bioscience Engineering, Ghent University, 9000 Ghent, Belgium
[2] Vandemoortele Centre 'Lipid Science and Technology', Faculty of Bioscience Engineering, Ghent University, 9000 Ghent, Belgium
* Correspondence: filip.vanbockstaele@ugent.be

Supplementary materials

Table S1. Composition of the fully hydrogenated TAG and MAG hardstock (in %).

	TAG hardstock	MAG hardstock
C16:0	5.3	5.3
C18:0	90.4	90.8
C20:0	1.7	2
Total SAFA	98.5	99.4
Monoglycerides	-	97.5
Triglycerides	98.8	-

Table S2. Melting temperature (in °C) of the dynamically produced oleogels as function of the storage time. Superscripts a-d indicate significant differences ($p<0.05$) between the different oleogels, while superscripts A-B indicate significant differences ($p<0.05$) as function of the storage time.

Storage (weeks)	Dy-M0-T100	Dy-M25-T75	Dy-M50-T50	Dy-M75-T25	Dy-M100-T0
0	$62.4 \pm 0.1^{a,A}$	$62.3 \pm 0.5^{a,A}$	$64.0 \pm 1.5^{a,A}$	$66.7 \pm 4.3^{a,b,A}$	$74.0 \pm 0.5^{b,A}$
1	$64.3 \pm 0.4^{a,B}$	$63.4 \pm 0.5^{a,A}$	$64.7 \pm 0.4^{a,A}$	$69.5 \pm 1.1^{b,A}$	$73.6 \pm 1.1^{b,A}$
4	$62.0 \pm 0.4^{a,b,A}$	$61.0 \pm 0.2^{a,B}$	$62.6 \pm 0.2^{b,A}$	$69.2 \pm 0.4^{c,A}$	$73.4 \pm 0.3^{d,A}$

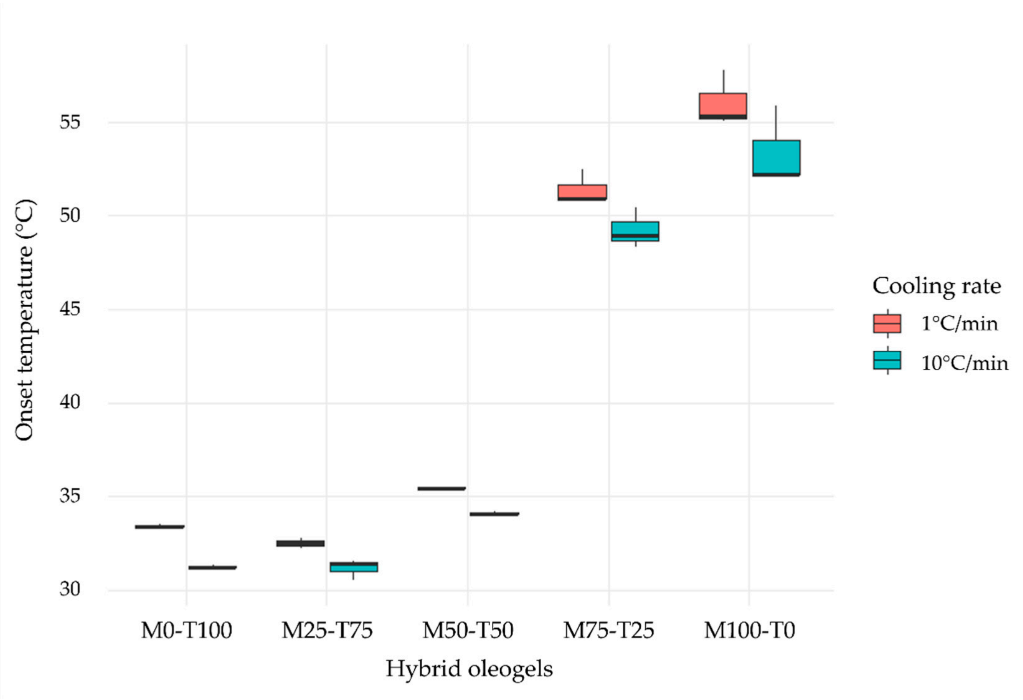

Figure S1. Onset temperature of crystallization for the slow and fast cooled hybrid oleogels. The boxplots represent the distribution of the onset temperature when crystallized at 1°C/min and 10°C/min.

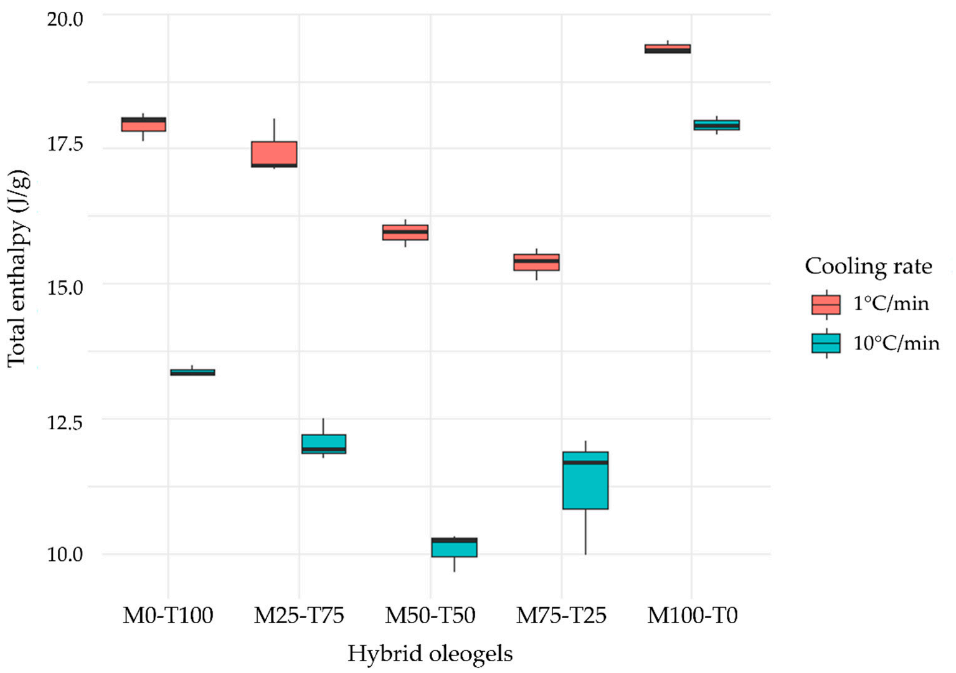

Figure S2. Total enthalpy of the slow and fast cooled hybrid oleogels. The boxplots represent the distribution of the enthalpy when crystallized at 1°C/min and 10°C/min.

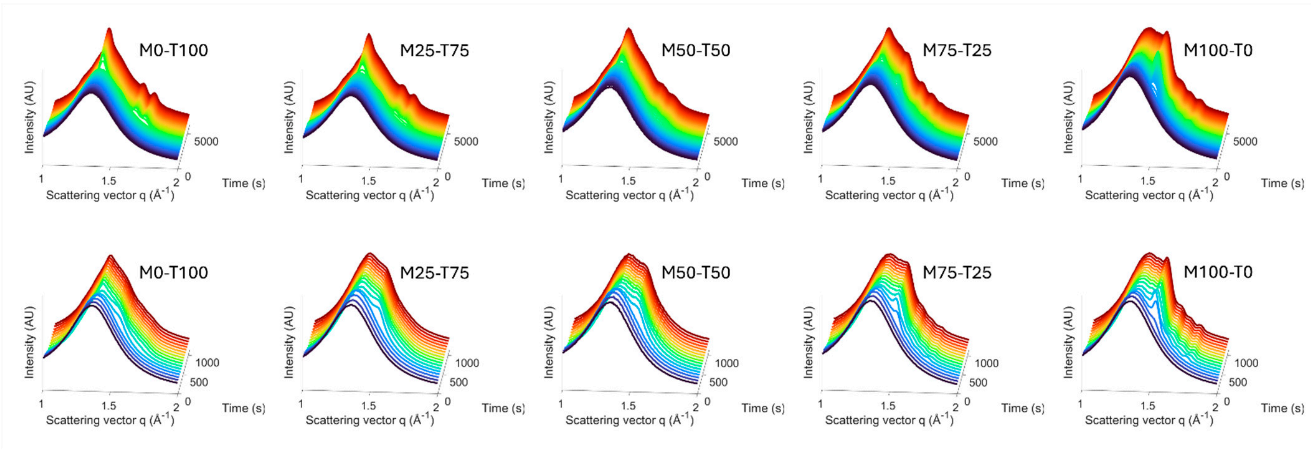

Figure S3. Evolution of the WAXS profile as function of the crystallization time upon cooling till 0°C at 1°C/min (top) and 10°C/min (bottom).

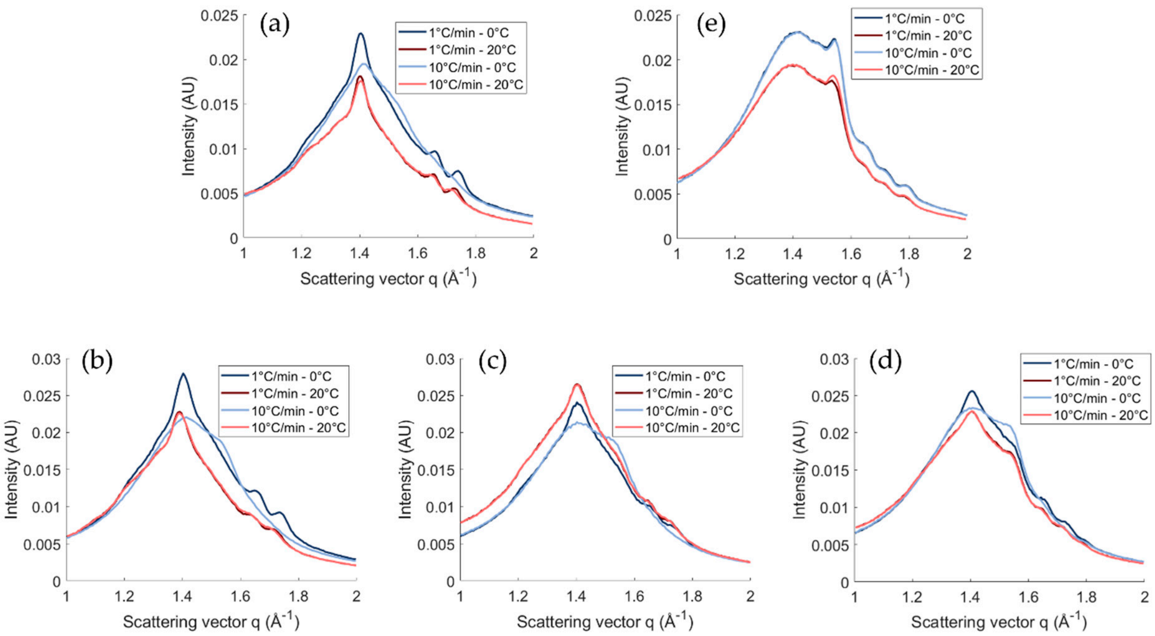

Figure S4. Final WAXS spectrum at the end of the isothermal time at 0°C and 20°C upon crystallization at 1°C/min and 10°C/min for (a) Dy-M0-T100, (b) Dy-M25-T75, (c) Dy-M50-T50, (d) Dy-M75-T25, (e) Dy-M100-T0.

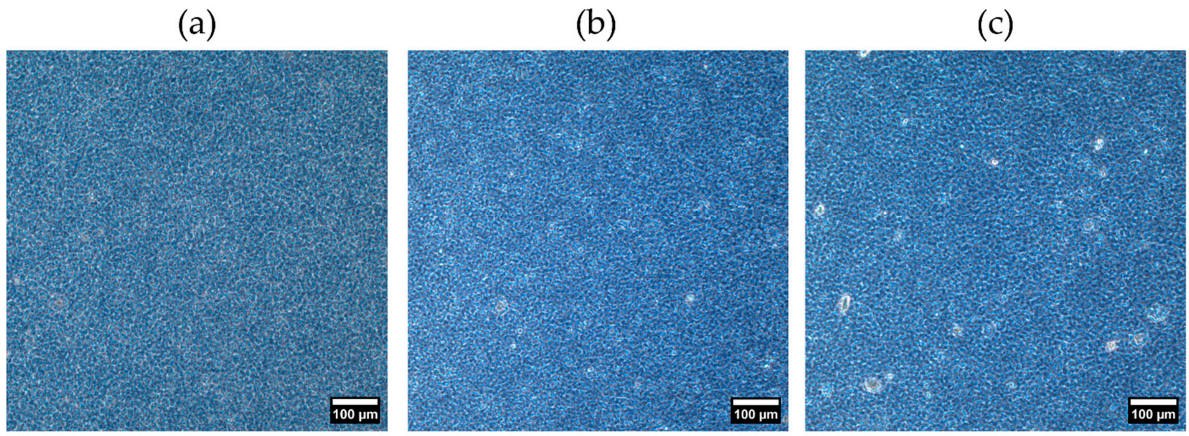

Figure S5. Phase contrast microscopy images of Dy-M0-T100 upon storage of 1 day (a), 1 week (b) and 4 weeks (c) at 20°C.

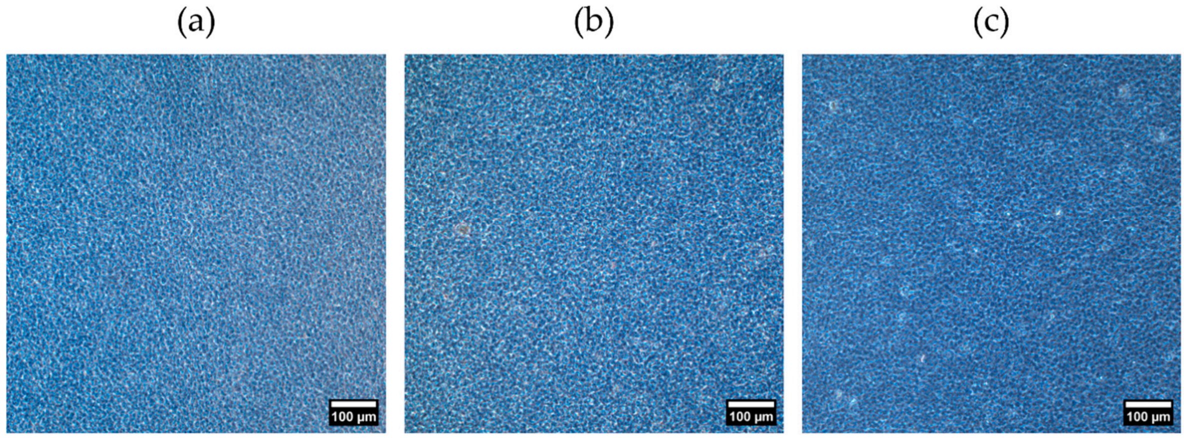

Figure S6. Phase contrast microscopy images of Dy-M25-T75 upon storage of 1 day (a), 1 week (b) and 4 weeks (c) at 20°C.

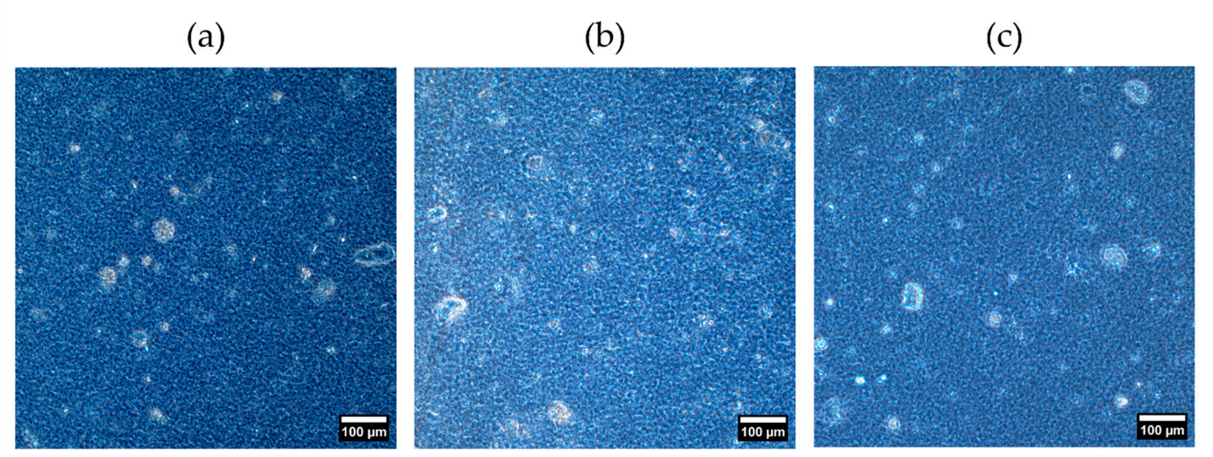

Figure S7. Phase contrast microscopy images of Dy-M50-T50 upon storage of 1 day (a), 1 week (b) and 4 weeks (c) at 20°C.

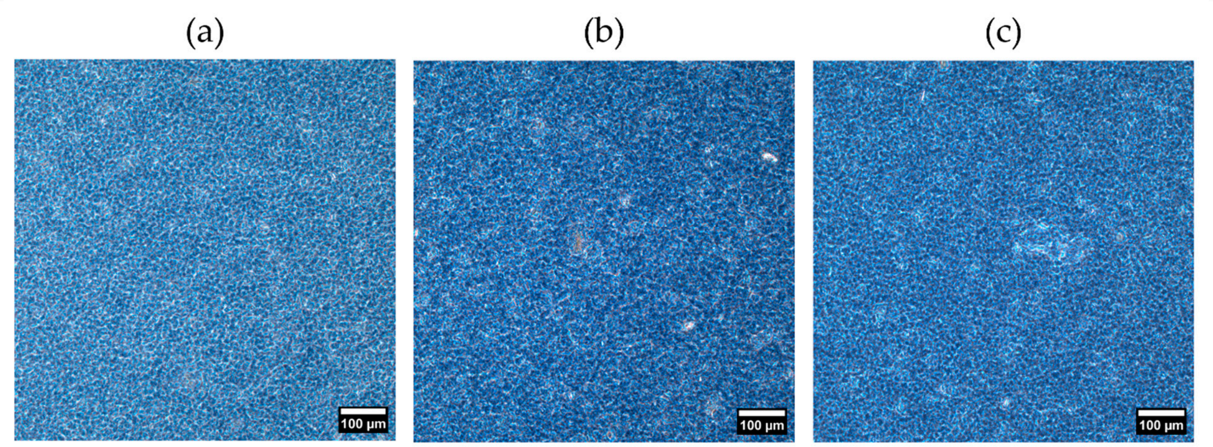

Figure S8. Phase contrast microscopy images of Dy-M75-T25 upon storage of 1 day (a), 1 week (b) and 4 weeks (c) at 20°C.

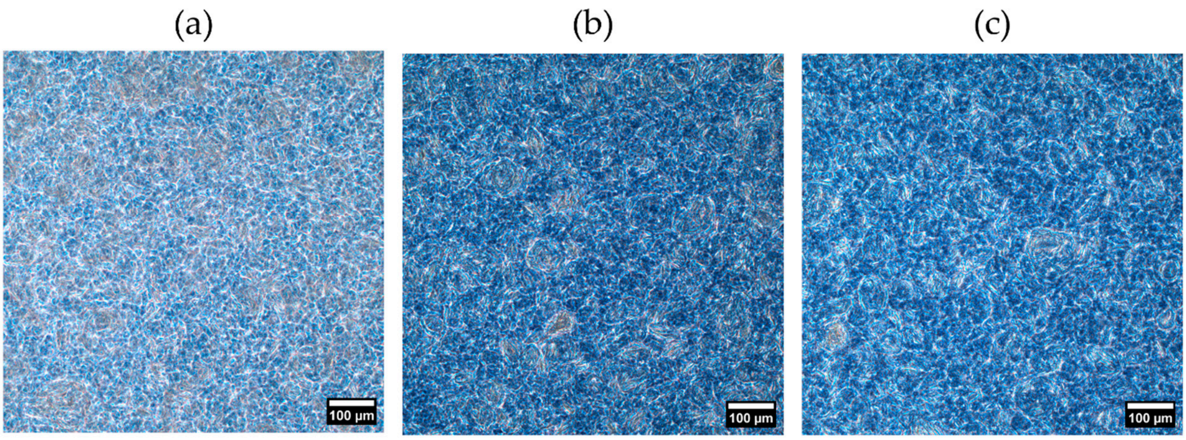

Figure S9. Phase contrast microscopy images of Dy-M100-T0 upon storage of 1 day (a), 1 week (b) and 4 weeks (c) at 20°C.
